# A novel fusion framework of deep bottleneck residual convolutional neural network for breast cancer classification from mammogram images

**DOI:** 10.3389/fonc.2024.1347856

**Published:** 2024-02-22

**Authors:** Kiran Jabeen, Muhammad Attique Khan, Mohamed Abdel Hameed, Omar Alqahtani, M. Turki-Hadj Alouane, Anum Masood

**Affiliations:** ^1^ Department of Computer Science, HITEC University, Taxila, Pakistan; ^2^ Department of Computer Science and Mathematics, Lebanese American University, Beirut, Lebanon; ^3^ Department of Computer Science, Faculty of Computers and Information, Luxor University, Luxor, Egypt; ^4^ College of Computer Science, King Khalid University, Abha, Saudi Arabia; ^5^ Department of Physics, Norwegian University of Science and Technology, Trondheim, Norway

**Keywords:** breast cancer, healthcare, bottleneck residual blocks, deep learning, fusion, optimization

## Abstract

With over 2.1 million new cases of breast cancer diagnosed annually, the incidence and mortality rate of this disease pose severe global health issues for women. Identifying the disease’s influence is the only practical way to lessen it immediately. Numerous research works have developed automated methods using different medical imaging to identify BC. Still, the precision of each strategy differs based on the available resources, the issue’s nature, and the dataset being used. We proposed a novel deep bottleneck convolutional neural network with a quantum optimization algorithm for breast cancer classification and diagnosis from mammogram images. Two novel deep architectures named three-residual blocks bottleneck and four-residual blocks bottle have been proposed with parallel and single paths. Bayesian Optimization (BO) has been employed to initialize hyperparameter values and train the architectures on the selected dataset. Deep features are extracted from the global average pool layer of both models. After that, a kernel-based canonical correlation analysis and entropy technique is proposed for the extracted deep features fusion. The fused feature set is further refined using an optimization technique named quantum generalized normal distribution optimization. The selected features are finally classified using several neural network classifiers, such as bi-layered and wide-neural networks. The experimental process was conducted on a publicly available mammogram imaging dataset named INbreast, and a maximum accuracy of 96.5% was obtained. Moreover, for the proposed method, the sensitivity rate is 96.45, the precision rate is 96.5, the F1 score value is 96.64, the MCC value is 92.97%, and the Kappa value is 92.97%, respectively. The proposed architectures are further utilized for the diagnosis process of infected regions. In addition, a detailed comparison has been conducted with a few recent techniques showing the proposed framework’s higher accuracy and precision rate.

## 1 Introduction

The most frequent tumor in women worldwide is breast cancer (1). Breast cancer ranks as the second most prevalent ailment among women across the globe. In the year 2022, more than 2.5 million women experienced breast cancer screening, and tragically, approximately 6.6% of them yielded to the disease. Breast cancer originates from abnormal cell proliferation within the breast tissue, often leading to the formation of a breast tumor and the potential spread of cancer to other parts of the body (2). Cancerous tumors are referred to as malignant because they hinder normal body functions and push out healthy tissue (3).

In contrast, benign tumors are noncancerous, since they do not spread to other parts of the body or have the ability to develop more tumors (4). Numerous imaging modalities have been created to help lower the death rate associated with breast cancer and to assist in the early detection and treatment of the disease (5). Breast exams, mammograms, and biopsy are just a few of the numerous examinations used in the detection and diagnosis of breast cancer (6). The more popular method for detecting breast cancer is mammography (7). An effective diagnostic methodology is essential for the timely diagnosis of such malignancy to increase survival (8).

Breast cancer imaging is crucial in lowering this unacceptably high mortality rate. Early detection of breast cancer allows for quicker treatment and higher survival rates than late-stage detection, which is why screening programs have been established (9). One of the significant reasons of death for women globally is breast cancer. Early detection and treatment are the best strategies to stop this cancer from spreading (10). Breast cancer imaging methods are also crucial for assessing and monitoring cancer treatment (11). The best, most dependable, and most cost-effective way for finding early indications of breast cancer is still mammography screening. To see anomalies, radiologists must carefully review mammography images (12). Consequently, the Medical Committee advises women to undergo widespread early mammography screening (13). Women aged 40 and above should undergo an annual mammogram (14).

Recently, computer-aided diagnosis (CAD) systems have performed a vital role in medical imaging, especially for breast cancer diagnosis, to minimize the operator-dependent workload of radiologists when interpreting digital mammography images (15). The purpose of a CAD system is to correctly classify the malignant and benign images, as 65%–90% of images belong to benign cancer. The challenges that increase the false positive rate are masses, architectural distortions, microcalcifications, and asymmetry (16). The diagnosis of microcalcifications has also been clinically authorized for effective CAD systems. Therefore, CAD solutions for breast masses generate a lot of scientific interest (12). Radiologists can identify and distinguish between normal and diseased tissues with CAD systems (17).

Deep learning technology has recently gained widespread adoption in the medical sector. This adoption is driven by the significant patient load and the pressing requirement to enhance the accuracy of pathology diagnosis, particularly in the context of detecting and classifying breast lesions. This technology supports physicians’ diagnostic efforts (18, 19). With convolutional neural networks (CNNs), the current deep learning algorithms have shown excellent performance in detecting and segmenting tumors in medical images (20). Deep-learning-based algorithms have demonstrated satisfactory performance in various computer vision applications, such as image classification, medical diagnosis, scene identification, disease prediction, and healthcare analysis (21). A few CAD systems collected straight features from images without doing segmentation or preprocessing work. This phase has the benefit of quick computing, but it has the drawback of extracting redundant and unnecessary information from the image’s noisy areas (22). The most significant information is found in the deeper layer, which is immediately computed into features in deep learning (23). However, the training dataset size, the choice of hyperparameters, and the cross-validation value all affect how well deep learning models perform. A deep learning model may have convolutional, ReLu, max pooling, and fully connected hidden layers. In deep learning, the softmax layer functions as a classifier. Several methods for diagnosing and classifying breast cancer have been presented in the literature and have increased accuracy rates. However, they employed the transfer learning idea and concentrated on the pre-trained models (i.e., VGG16 (24), Alexnet (25), ResNet (24), MobileNet (26), and EfficienctNet (27)). For training purposes, those models need a large and well-balanced dataset of images; however, the publicly accessible breast cancer dataset is insufficient (28). In addition, the extraction of irrelevant feature extraction decreased the classification accuracy and increased the computation time (second).

As a result, a unique model that can train on a limited number of images and offer improved classification accuracy is frequently needed. This article proposes a novel deep bottleneck residual convolutional neural network fusion architecture for diagnosing and classifying breast cancer. The suggested technique additionally uses an optimization algorithm to increase accuracy and decrease computing time. The following are the principal contributions of this work:

- We proposed a novel bottleneck residual convolutional neural network (CNN) architecture with three parallel residual blocks and 76 hidden layers, including convolutional, average pooling, and fully connected.
- We proposed a novel single-path bottleneck residual CNN architecture with four residual blocks and 60 hidden layers.
- The hyperparameters of the proposed models are initialized using a Bayesian Optimization instead of manual alteration.
- A kernel-based canonical correlation analysis and entropy technique is proposed for the extracted deep features fusion. The fused feature set is further refined using an optimization approach named generalized normal distribution optimization.
- A detailed comparative analysis has been performed for the proposed method. In addition, a detailed ablation study has been performed.

## 2 Literature review

Despite researchers having created numerous feature extraction and disease classification strategies, there is still an opportunity for improvement (29). The researchers provided deep-learning methods for breast cancer diagnosis and classification (30). A novel approach for mass classification tasks that simultaneously trains on texture and deep Convolutional Neural Network (CNN) representations was offered by Zhang et al. (16). The CNN-based classification was merged with rotation-invariant features of the maximum response filter bank. The fusion procedure addressed CNN’s shortcomings in capturing mass attributes after the reduction technique was implemented. The mini-MIAS and INbreast combined dataset and other publicly available datasets like CBIS-DDSM were used to train this model. The CBIS-DDSM dataset was used to test the reduction strategy and fusion, and the results demonstrated that this method exceeded expectations other models regarding accuracy (94.30%). You-Only-Look-Once (YOLO) was the foundation for the end-to-end system introduced by Baccouche et al. (18). This approach could classify and identify breast abnormalities in complete mammograms that could be of concern at the same time. The algorithm began by preprocessing the raw pictures, identifying the abnormal regions as breast lesions, and then classifying the lesions’ pathology as either masses or calcifications. Two publicly available datasets were used to assess the model: one had 2,907 mammograms from the CBIS-DDSM’s Curated Breast Imaging Subset, and the other contained 235 mammograms from the INbreast database. The assessment procedure also utilized a privately assembled dataset consisting of 487 mammograms. Furthermore, a fusion model method was suggested to improve detection and classification accuracy.

PatchSample decomposition is a revolutionary technique Harris et al. (31) devised for learning sparse approximations and making classification judgments. In contrast to BlockBoost, the prior method, PatchSample, builds larger dictionaries that encompass a wider variety of visual data from every point inside the region of interest (ROI) and spatially specific information. Notably, a combined dataset of mammograms from two separate providers was utilized to train and examine the approach. The experimental results described that applying PatchSample decomposition to a combined dataset consisting of the MLO view regions of interest from both the MIAS and CBIS-DDSM datasets could result in classification accuracy (ACC) of up to 67.80% and (AUC) of 73.21%. Deep transfer learning techniques were used by TİRYAKİ et al. (32) to classify calcification diseases and breast cancer masses. Convolutional neural networks were trained and tested on a dataset of 3,360 patches taken from the CBIS-DDSM and (DDSM) mammography databases. Resnet50, NASNet, Xception, and EfficientNet-B7 network backbones were used to apply transfer learning. The Xception network produced the best categorization results out of all of them. In particular, an area under the curve (AUC) of 0.9317 was obtained for the five-way classification problem using the original CBIS-DDSM test data. A fresh architecture for a capsule network was proposed by Soulami et al. (33), which significantly reduced the original capsule network’s computing time by a factor of 6.5. This improvement made training breast mass areas of interest (ROIs) on less expensive GPUs possible. The proposed architecture was refined by adding data augmentation techniques and changing the number of kernels and capsules. The higher effectiveness of our capsule-based approach in the one-stage classification of suspicious breast masses was demonstrated by evaluation findings across four categories of breast density. The model’s accuracy in binary classification—which discerns between normal and abnormal masses—was 96.03%. The model had a 77.78% accuracy rate in the multi-class classification of breast masses into benign, malignant, and normal categories.

A novel multi-stage transfer learning (TL) technique for differentiating between benign and malignant mammographic breast masses was presented by Ayana et al. (34). Images of cancer cell lines and pre-trained models from ImageNet were employed in this technique. The three publicly available datasets used to train the model were DDSM, MIAS, and INbreast. Furthermore, training was conducted using a composite dataset that included photos from all three sources. The average area under the curve for the DDSM, MIAS, INbreast, and mixed datasets was 1, 0.9993, 0.9994, and 0.9998, respectively, according to the fivefold cross-validation results. Using the MIAS and INbreast databases, Aslan et al. (35) sorted mammography images into normal, benign, and malignant categories. After the photos were preprocessed, the processed images were fed into two different end-to-end deep networks. While the second network was intended to have a hybrid structure that combined both the CNN and Bidirectional Long Short-Term Memories (BiLSTM), the first network was composed entirely of a CNN. For the MIAS dataset, the first and second hybrid architectures produced classification accuracy of 97.56% and 97.60%, respectively. A deep learning algorithm-based training technique that enhances edge detail and reduces false positives for automated early detection of breast cancer was presented by Devendhiran et al. (36). The recommended approach combines an optimization strategy with a CNN to produce a classification model for the identification of breast cancer. Utilizing a hybrid approach, the advantages of the Whale Optimization Algorithm (WOA) and the Marine Predators Algorithm (MPA) were merged to determine the optimal hyperparameter values for the CNN framework. The proposed technique leveraged a pre-trained convolutional neural network model called Inception v3 and DenseNet. By comparing the attainment of two hybrid models, the research showed that the MPA-WOA with DenseNet attained an exactness rate of 94% and 95% for the CBIS-DDSM and MIAS datasets, respectively.

This literature review discusses many deep-learning algorithms for breast cancer diagnosis and classification. Researchers have looked into techniques including using You-Only-Look-Once (YOLO) for end-to-end systems, combining texture and deep Convolutional Neural Network (CNN) representations, and introducing PatchSample decomposition for sparse approximations. Several network backbones, including Resnet50, NASNet, Xception, and EfficientNet-B7, have been used in transfer learning approaches to categorize masses of breast cancer and calcification illnesses. A brand-new capsule network design showed decreased processing times and increased efficiency. When distinguishing between benign and malignant mammographic breast masses, multi-stage transfer learning methods that used pre-trained models and composite datasets demonstrated encouraging results. High classification accuracy was attained by hybrid architectures that combined Bidirectional Long Short-Term Memories (BiLSTM) with CNN. Furthermore, on datasets like CBIS-DDSM and MIAS, a deep learning algorithm that combined optimization algorithms and pre-trained models, including Inception v3 and DenseNet, revealed efficient automated early detection of breast cancer with high accuracy rates. Overall, these findings demonstrate how the field of breast cancer diagnosis is changing through creative methods and point to the possibility of further advancements in precision and efficiency.

## 3 Proposed methodology

**Figure 1:**
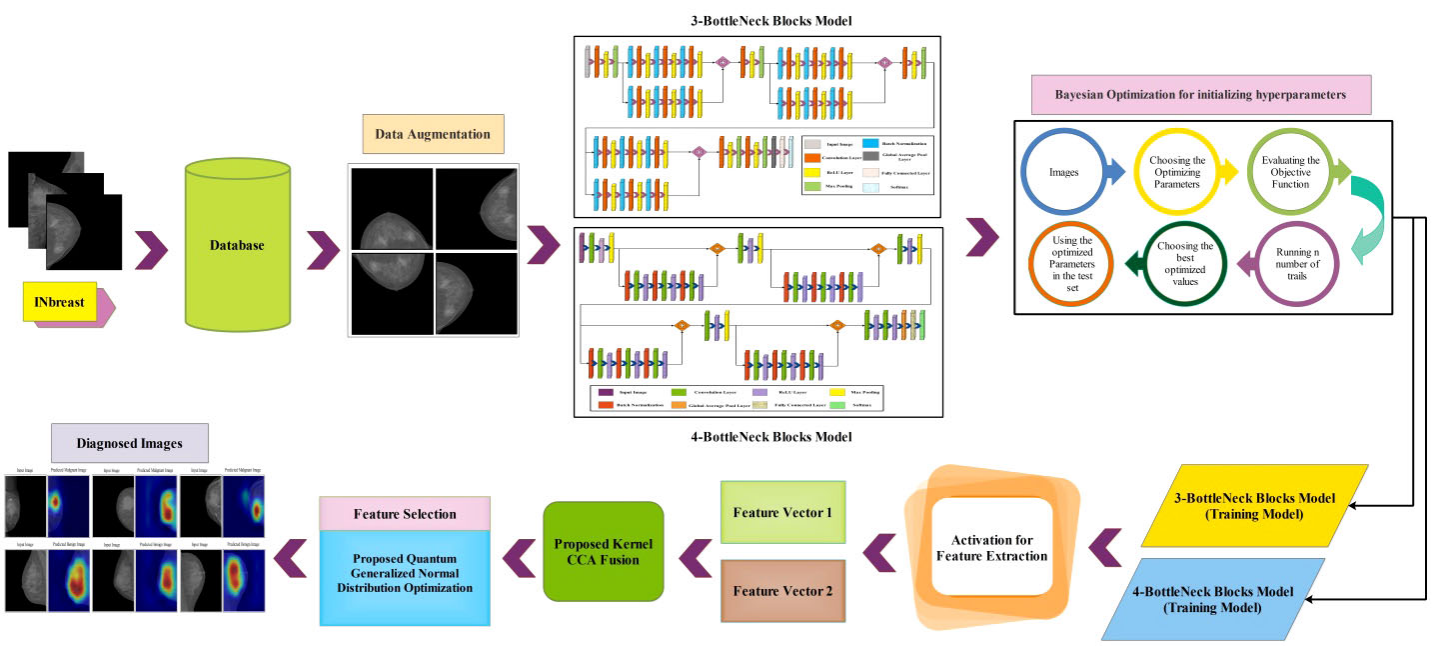
Proposed methodology for breast cancer classification and diagnosis.

In this section, a proposed methodology for breast cancer classification has been performed using mammogram images. **Figure 1** presents the proposed method, which consists of several steps. The INbreast dataset was employed in the first step, and the data augmentation process was performed using the traditional technique to enhance the quantity of dataset and to improve the accuracy of the model. Two novel CNN architectures named the three-block bottleneck residual model and the four-block bottleneck residual model have been proposed. Bayesian Optimization has been employed to initialize hyperparameters throughout the training phase. Features are extracted from the global average pooling layer and fused using a novel technique canonical correlation analysis-based technique. Afterward, a quantum generalized normal distribution optimization algorithm was implemented, and the best features were selected. In the next step, neural network classifiers are utilized for the classification process. Finally, the tumor diagnosis has been performed for the malignant tumor using an explainable AI technique.

### 3.1 Dataset of the proposed work

**Figure 2:**
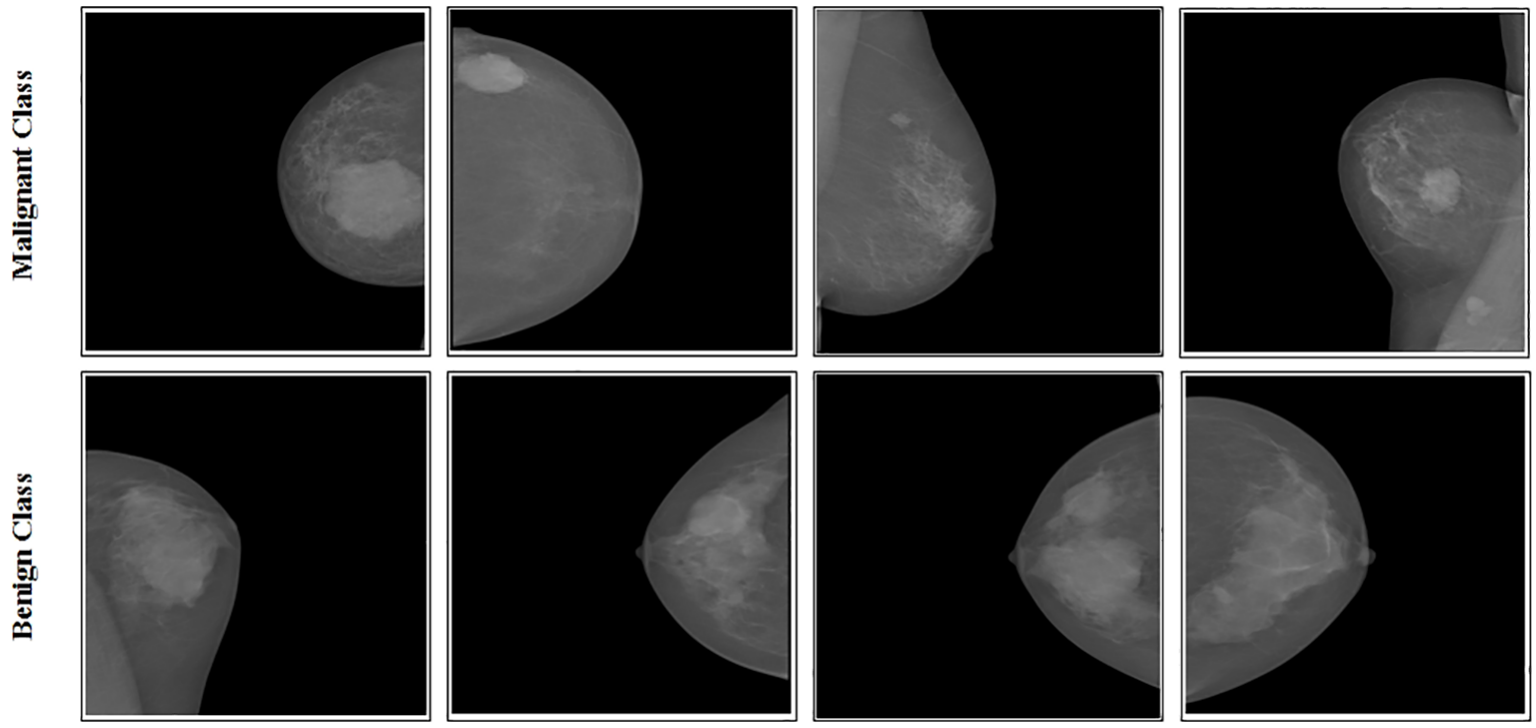
Samples images of INbreast dataset.

INbreast data were gathered at Centro Hospitalar de S. Joao [CHSJ], Breast Centre, Porto, a university hospital in Portugal. The study (cases) covered a total of 115 people. A total of 410 mammograms with CC and MLO images were performed (37). The INbreast dataset includes two categories: benign and malignant. **Figure 2** displays sample images from this dataset, containing 410 images belonging to 115 patients. These images come in two different sizes: 2,560 × 3,328 pixels and 3,328 × 4,084 pixels. In the experimental process, 108 mammogram images of masses were used (38).

### 3.2 Proposed bottleneck layered model

In a layered model, a “bottleneck” or “bottleneck layer” usually refers to a particular architectural element frequently employed in deep neural networks, especially in CNNs. A bottleneck layer’s goal is to lower the feature map’s dimensionality while retaining crucial data, which might result in more effective and computationally less expensive models. A particular kind of neural network building block is called a bottleneck block. There are three main parts to each bottleneck block.

*1×1 Convolutional layer.* This layer looks at a tiny portion of the input data, similar to a small filter. It employs small filters with a 1×1 pixel size because it is called “ 1×1 .” By reducing the amount of characteristics or channels in the data, this layer helps conserve computational resources.

*3×3 Convolutional layer*. The 3×3 convolutional layer employs larger 3×3 filters to detect intricate patterns and features within the data. It functions on the decreased number of channels generated by the preceding 1×1 convolutional layer.

*1×1 Convolutional layer*. The 1×1 convolutional layer comes after the 3×3 convolution, performing another round of 1×1 convolution. This additional step increases the number of features, thus revitalizing and enriching the data representation.

#### 3.2.1 Three-block bottleneck layered model

In this work, a three-block bottleneck layered model has been proposed for the classification of breast cancer into malignant and benign. Each block follows the arrangement described above.

**Figure 3:**
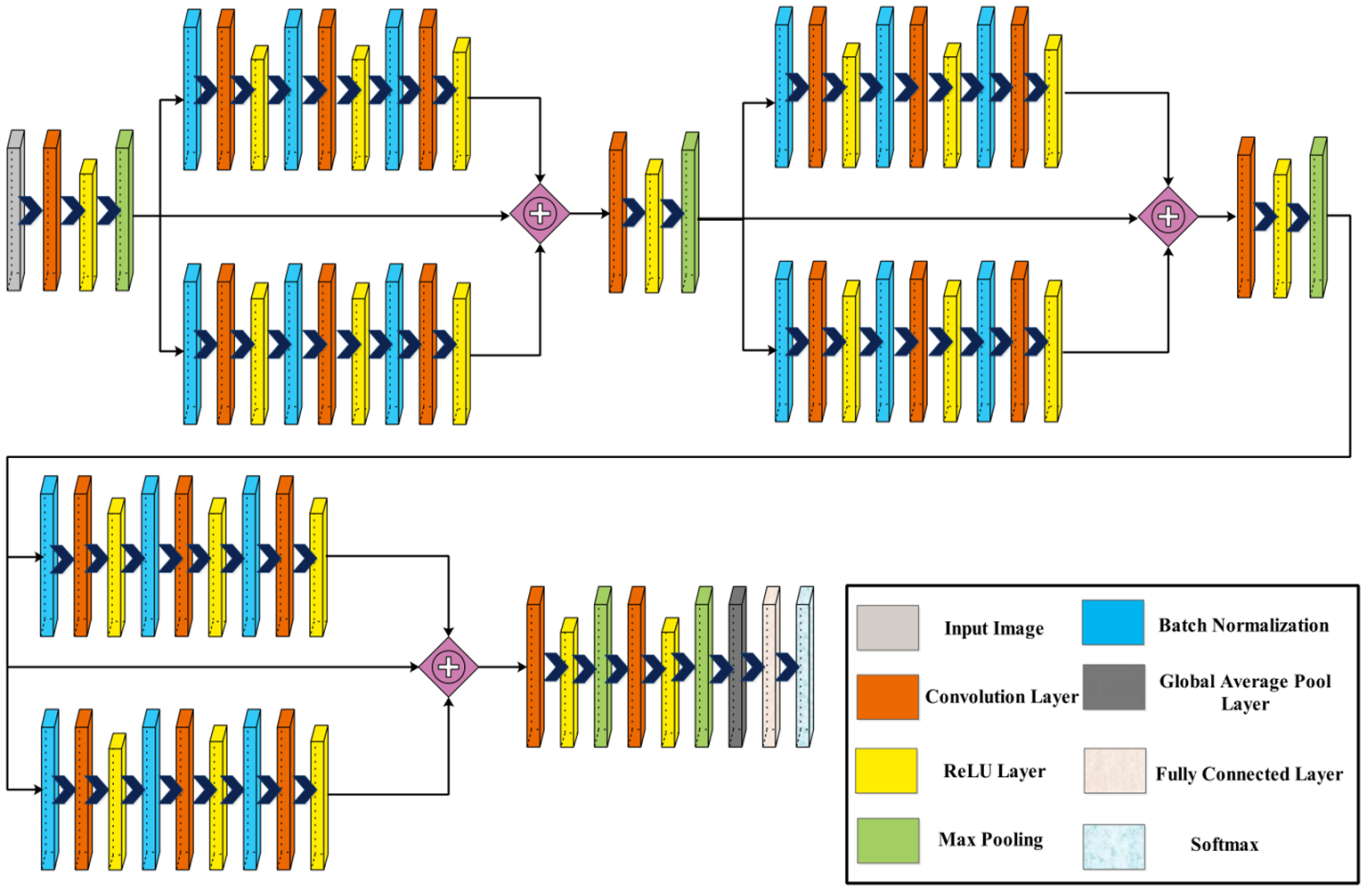
Proposed three-block bottleneck layered model architecture.

**Figure 3** displays the architecture of the three-block bottleneck layered model. In this network architecture, the initial input dimensions are 227×227×3 , subsequently processed by the first convolutional layer with a depth of 32, a filter size of 3×3 , a stride of 2, and a ReLU activation layer. Following this, a maxpooling layer with a 3×3 filter and a stride of 1 is applied. After that, two bottleneck blocks are added in parallel, each including a batch normalization layer, convolution layer of depth 64, filter size of 1×1 , stride of 1, and ReLU activation layer. Then, a second batch normalization layer is added to this block, followed by a convolution layer of depth of 64, filter size of 3×3 , stride of 1, and a ReLU activation layer. After that, a convolution layer is added with depth of 64, filter size of 3×3 , and stride two, followed by the ReLU activation layer and max pooling layer.

Next, two blocks are appended in parallel, each comprising a batch normalization layer, followed by a convolution layer and a ReLU activation layer. A second batch normalization layer is added, followed by a convolution layer and a ReLU activation layer. Similarly, a third batch normalization layer is introduced, succeeded by a convolution layer and another ReLU activation layer. This sequence is repeated once more with the addition of two more blocks.

**Figure 4:**
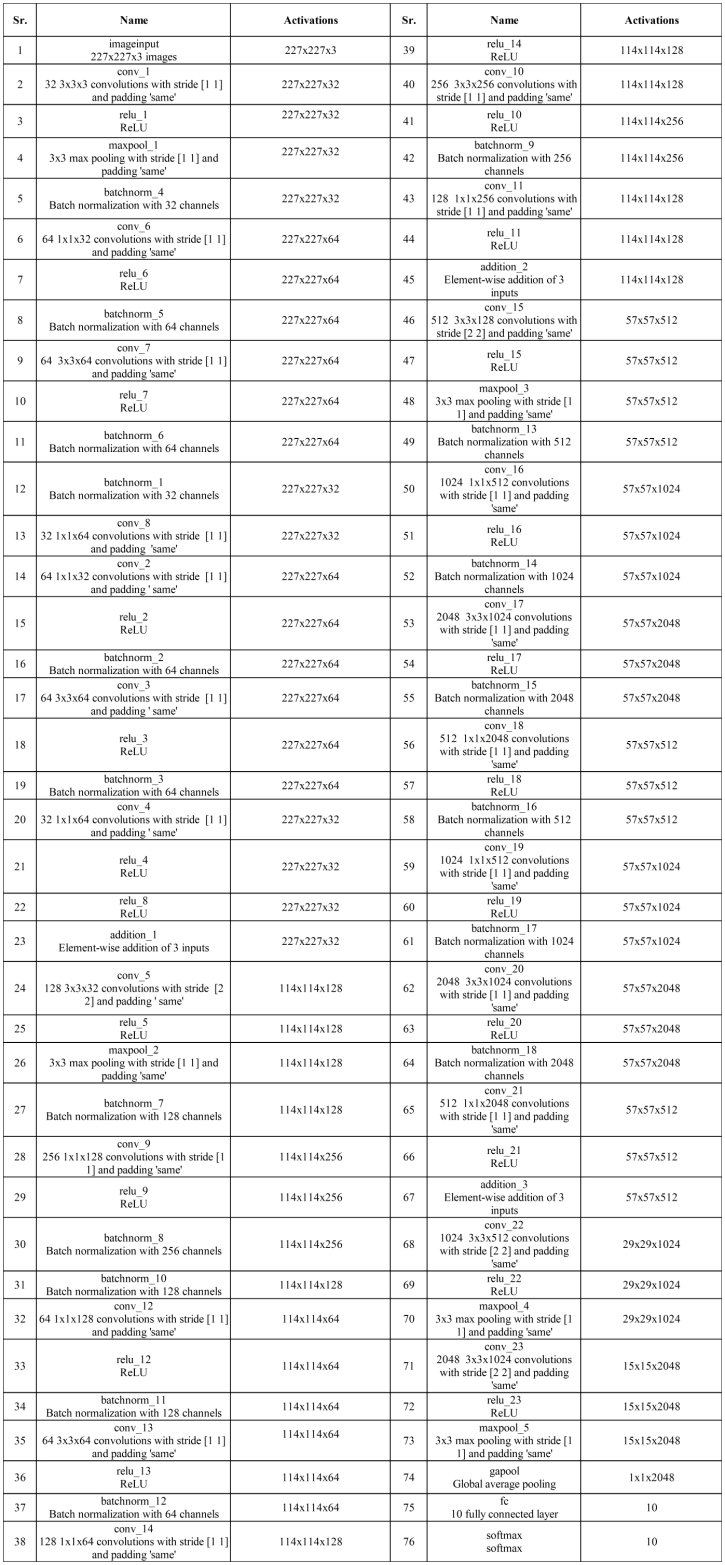
Proposed three-block bottleneck layered detailed architecture.

Subsequently, a convolution layer with a depth of 1,024, a filter size of 3×3 , and a stride of 2 is incorporated, followed by a ReLU activation layer. Following this, another convolution layer is introduced with a depth of 2,048, a filter size of 3×3 , and a stride of 2, followed by a ReLU activation layer. Finally, the network concludes with a global average pool layer, a fully connected layer, and a softmax layer. **Figure 4** shows the detailed architecture of the three-block bottleneck layered model. The number of trained parameters of three-block bottleneck residual model is 15.9M.

#### 3.2.2 Four-block bottleneck layered model

**Figure 5:**
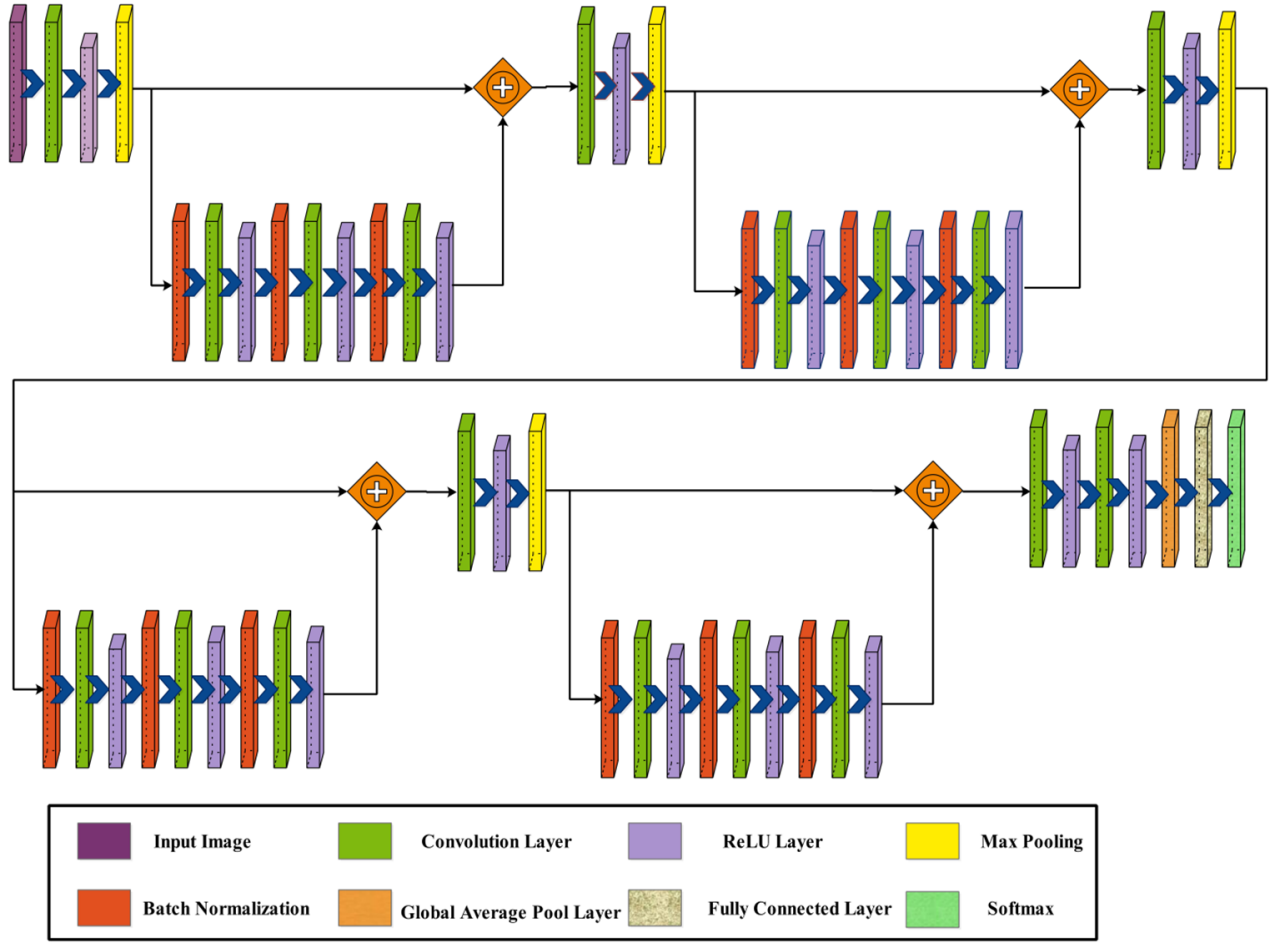
Proposed four-block bottleneck layered model architecture.

The phrase “four bottleneck blocks” refers to how this neural network is built by stacking three of these bottleneck blocks. Each block follows the above-described structure. **Figure 5** shows the proposed architecture of four-block bottleneck layered.

In this network architecture, the initial input dimensions are 227×227×3 , subsequently processed by the first convolutional layer with a depth of 32, a filter size of 3×3 , a stride of 2, and a ReLU activation layer. Following this, a maxpooling layer with a 3×3 filter and a stride of 1 is applied. After that, a bottleneck block is added, including a batch normalization layer, convolution layer of depth of 64, filter size of 1×1 , stride of 1, and ReLU activation layer. Then, a second batch normalization layer was added to this block, followed by a convolution layer of depth of 64, filter size of 3×3 , stride of 1, and a ReLU activation layer.

Following that, a block is added, which consists of a batch normalization layer, followed by a convolution layer and a ReLU activation layer. Subsequently, a second batch normalization layer is introduced, followed by a convolution layer and a ReLU activation layer. Similarly, a third batch normalization layer is integrated, with a convolution layer and another ReLU activation layer. This sequence is repeated once more with the addition of two more blocks.

**Figure 6:**
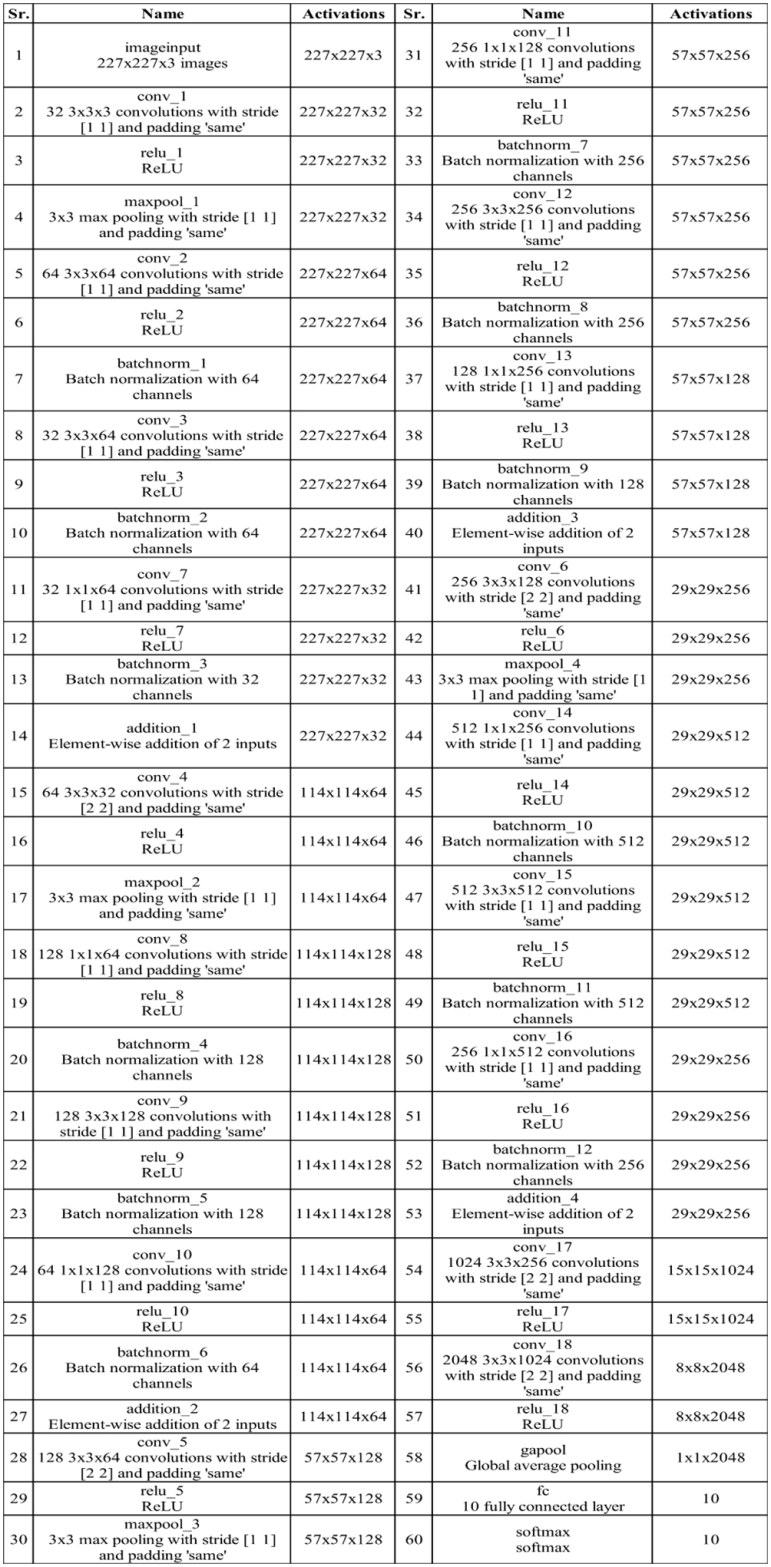
Proposed four-block bottleneck layered model detailed architecture.

Afterward, a convolution layer is introduced with a depth of 1,024, a filter size of 3×3 , and a stride of 2, followed by a ReLU activation layer. Following this, another convolution layer includes a depth of 2,048, a filter size of 3×3 , and a stride of 2, followed by a ReLU activation layer. To conclude, the network is finalized by adding a global average pool layer, a fully connected layer, and a softmax layer. **Figure 6** shows the detailed architecture of the four-block bottleneck layered model. In this figure, the details of layers and weights are described. The number of trained parameters of the four-block bottleneck residual model is 25.1M.

#### 3.2.3 Training models and features extraction

The proposed model training process has been described in this section. A 50:50 approach has been opted in the training process, meaning that 50% of the images have been employed, and the remaining images have been utilized for the testing method. Several hyperparameters have been selected in the training process by employing the Bayesian Optimization algorithm (39), such as a learning rate value of 0.000241, a momentum value of 0.776, epochs of 50, mini-batch size of 64, and stochastic gradient descent as an optimizer. After that, both models were trained and later utilized for feature extraction. The number of extracted features for both model is 2,048. The testing images have been implemented for the testing feature extraction. The global average pooling layer has been selected for the feature extraction. For both models, 2,048 features are extracted and mathematically presented as follows.

Consider two trained proposed deep learning architectures, such as ${\text{Δ}}_{1}\in$ proposed three-block bottleneck architecture and ${\text{Δ}}_{2}\in$ proposed four-block bottleneck architecture, respectively. The global average pool layer has been selected as a feature layer in both models and defined by ${\text{L}}_{1}$ for ${\text{Δ}}_{1}$ and ${\text{L}}_{2}$ for ${\text{Δ}}_{2}$ , respectively. Hence, the activation has been performed as follows:


$${\tilde{F}}_{1}\left(k\right)=A\left({\text{Δ}}_{1},{\text{L}}_{1}\right),\;{\tilde{F}}_{1}\left(k\right)\in \mathbb{R}$$

$${\tilde{F}}_{2}\left(k\right)=A\left({\text{Δ}}_{1},{\text{L}}_{2}\right),\;{\tilde{F}}_{2}\left(k\right)\in \mathbb{R}$$

where ${\tilde{F}}_{1}\left(k\right)$ and ${\tilde{F}}_{2}\left(k\right)$ are feature matrix of dimensional N×2,048 and N×2,048 , respectively. In the next stage, an enhanced fusion technique is employed to fuse the extracted features.

### 3.3 Proposed features fusion

A novel features fusion technique has been proposed in this work and presented under this section for the fusion of ${\tilde{F}}_{1}\left(k\right)$ and ${\tilde{F}}_{2}\left(k\right)$ . A kernel-based canonical correlation analysis and entropy technique have been implemented for feature fusion. The original CCA technique (40) is a linear algorithm that can potentially reveal the linear correlation between two feature vectors. However, there is a non-linearity problem among them; therefore, we employed a kernel-based CCA technique for the fusion process.

Consider two feature matrix sets ${\tilde{F}}_{1}\left(k\right)$ and ${\tilde{F}}_{2}\left(k\right)$ , defined as ${\tilde{F}}_{1}={\left(u_{1},u_{2},\ldots ,\;u_{p}\right)}^{T}$ and ${\tilde{F}}_{2}={\left(v_{1},v_{2},\ldots ,\;v_{q}\right)}^{T}$ . If each variable has n sample points, then the matrix $U_{p\times n}=\left(u_{1},u_{2},\ldots ,\;u_{n}\right)$ and $V_{q\times n}=\left(v_{1},v_{2},\ldots ,\;v_{n}\right)$ are created. Suppose ψ maps the original extracted feature vector $U_{p\times n}$ into a high-dimensional feature space $F_{u}$ that is $\psi :u_{i}\to \psi \left(u_{i}\right)\in F_{u}$ ; φ map $V_{q\times n}$ into feature space $F_{v}$ that is $\varphi :v_{i}\to \psi \left(v_{i}\right)\in F_{v}$ ; therefore, two feature matrices $\psi \left(U\right)=\left[\psi \left(u_{1}\right),\psi \left(u_{2}\right),\;\ldots ,\;\psi \left(u_{n}\right)\right]$ and $\psi \left(V\right)=\left[\psi \left(v_{1}\right),\psi \left(v_{2}\right),\;\ldots ,\;\psi \left(v_{n}\right)\right]$ are designed.

The aim of kernel CCA is to find two basic vectors $\alpha_{\psi }$ and $b_{\varphi }$ in feature space such that the correlation coefficient between $Q=\alpha_{\psi }^{T}\psi \left(U\right)$ and $R=b_{\varphi }^{T}\varphi \left(V\right)$ is maximized, can be formulated by **Equations 1**, **2**.


(1) $$\rho_{Q,R}=\frac{\alpha_{\psi }^{T}\psi \left(U\right)\varphi {\left(V\right)}^{T}b_{\varphi }}{\sqrt{\alpha_{\psi }^{T}\psi \left(U\right)\psi {\left(U\right)}^{T}\alpha_{\psi }}\sqrt{b_{\varphi }^{T}\varphi \left(V\right)\varphi {\left(V\right)}^{T}b_{\varphi }}}$$

(2) $$\{\begin{array}{c} \alpha_{\psi }=\sum_{i=1}^{n}\xi_{i\;}\psi \left(u_{i}\right)=\psi \left(U\right)\xi \\ b_{\varphi }=\sum_{i=1}^{n}\eta_{i}\;\varphi \left(v_{i}\right)=\varphi \left(V\right)\eta \end{array}$$

By putting the value of $\alpha_{\psi }$ and $b_{\varphi }$ into $\rho_{Q,R}$ , we computed the following equation (**Equation 3**):


(3) $$\rho_{Q,R}=\frac{\xi^{T}\psi {\left(U\right)}^{T}\psi \left(U\right)\varphi {\left(V\right)}^{T}\varphi \left(V\right)\eta }{\sqrt{\xi^{T}\psi {\left(U\right)}^{T}\psi \left(U\right)\psi {\left(U\right)}^{T}\psi \left(U\right)\xi }\sqrt{\eta^{T}\varphi {\left(V\right)}^{T}\varphi \left(V\right)\varphi {\left(V\right)}^{T}\varphi \left(V\right)\eta }}$$

By employing the kernel trick, the inner product between $\psi \left(u_{i}\right)$ and $\varphi \left(u_{j}\right)$ can be replaced by a kernel function $K_{u}\left(u_{i},\;u_{j}\right)$ . In this work, we employed the Gaussian kernel function instead of the sigmoid function. Mathematically, this function is defined as follows is defined by **Equations 4**, **5**:


(4) $$K_{u}\left(u_{i},\;u_{j}\right)=\psi {\left(u_{i}\right)}^{T}\psi \left(u_{j}\right)$$

(5) $$K_{v}\left(v_{i},\;v_{j}\right)=\varphi {\left(v_{i}\right)}^{T}\varphi \left(v_{j}\right)$$

Hence, the two kernel matrices are obtained as follows by **Equations 6**, **7**:


(6) $${\left(K_{u}\right)}_{ij}=K_{u}\left(u_{i},\;u_{j}\right)$$

(7) $${\left(K_{v}\right)}_{ij}=K_{v}\left(v_{i},\;v_{j}\right)$$

Hence, the correlation between Q,R is defined as follows defined by **Equation 8**:


(8) $$\rho_{Q,R}=\frac{\xi^{T}K_{u}K_{v}\eta }{\sqrt{\xi^{T}K_{u}K_{u}\xi }\sqrt{\eta^{T}K_{v}K_{v}\eta }}$$

where $\xi^{T}K_{u}K_{u}\xi =1$ and $\eta^{T}K_{v}K_{v}\eta =1$ . Hence, finally, maximizing the correlation coefficient among ξ and η is defined as follows defined by **Equation 9**:


(9) $$\underset{\xi ,\eta }{\max}\xi K_{u}K_{v}\eta \;\;\text{s}.\text{t}.\;\xi^{T}K_{u}K_{u}\xi =\eta^{T}K_{v}K_{v}\eta =1$$

Based on the Lagrange multiplier method, the optimization problem is transformed as follows by **Equation 10**:


(10) $$\left[\begin{array}{c} \;\;\;\;\;\;\;\;K_{u}K_{v}\\ K_{v}K_{v}\;\;\;\;\;\;\;\; \end{array}\right]\left[\begin{array}{c} \xi \\ \eta \end{array}\right]=\lambda \left[\begin{array}{c} K_{u}K_{u}\;\;\;\;\;\;\\ \;\;\;\;\;\;\;\;K_{v}K_{v} \end{array}\right]\left[\begin{array}{c} \xi \\ \eta \end{array}\right]$$

The entropy is employed on the transformed matrix to handle the problem of uncertainty. The returned feature matrix K(u,v) is optimized using the quantum generalized normal distribution algorithm. The vector size of the fused feature is 1,168×4,096 .

### 3.4 Quantum generalized normal distribution optimization algorithm

A novel optimization algorithm called GNDO has recently been suggested by Zhang et al. (41). In this work, we improved the working of GNDO by employing the quantum mechanism. The GNDO is structured in a pretty simple way, and it intends to share information through both global and local exploration and exploitation. The present ideal location and mean position influence the generalized normal distribution model utilized for local exploitation. On the other hand, global exploration is associated with the selection of three individuals at random.

*Local exploitation.* Finding better answers inside a search space composed of everyone’s current placements is known as local exploitation. An optimal generalized normal distribution model can be created based on the link between the normal distribution and the individual distribution of the population. Mathematically, the optimization method is formulated by **Equations 11**–**27**.


(11) $$Z_{j}^{\tau }=\partial_{j}+{∁}_{j}\times \;CT,\;j=1,2,3,\ldots ,n$$

where the trailing vector $Z_{j}^{\tau }$ represents the  jth individual’s trajectory at time  τ , while $\partial_{j}\;$ denotes their generalized mean position, ${∁}_{j}$ signifies the generalized standard variance, and CT serves as the penalty factor. Furthermore, $\partial_{j}$ , ${∁}_{j}$ , and ∈ can be characterized as:


(12) $$\partial_{j}=\frac{1}{3}\left(V_{j}^{\tau }+V_{best}^{\tau }+K\right)$$

(13) $${∁}_{j}=\sqrt{\frac{1}{3}\;\left[{\left(V_{j}^{\tau }-\;\partial \right)}^{2}+{\left(V_{best}^{\tau }-\;\partial \right)}^{2}+{\left(K-\partial \right)}^{2}\right]}$$

(14) $$CT=\{\begin{array}{c} \sqrt{-log\left(\gamma_{1}\right)\;}\times \cos\left(2\pi \gamma_{2}\right),\;\;if\;x\leq y\\ \sqrt{-log\left(\gamma_{1}\right)}\times cos\left(2\pi \gamma_{2}+\pi \right),\;Otherwise \end{array}$$

where x , y , $\gamma_{1}$ , and $\gamma_{2}$ are randomly generated numbers within the range of 0–1, and $V_{best}^{\tau }$ represents the present best position. Additionally, K denotes the current population’s mean position, and it can be calculated using the following method:


(15) $$K=\frac{\sum_{j=1}^{n}V_{j}^{\tau }}{n}$$

Next, the roles of the three parameters utilized, namely $,\;\partial_{j}$ , ${∁}_{j}$ , and ∈ within the designed local exploitation strategy are explained.

- The generalized mean position, denoted as $\partial_{j}$ , plays a crucial role. The current best individual is represented as $V_{best}^{\tau }$ carries valuable insights about the global optimal solution. Consequently, the jth individual, $V_{j}^{\tau }$ , is attracted within the direction of the current best individual, $V_{best}^{\tau }$ , increasing its likelihood of discovering an improved solution. It is important to note that if $V_{best}^{\tau }$ becomes trapped in a local optimum, all individuals will continue to gravitate toward the direction of $V_{best}^{\tau }$ . This behavior can lead to premature convergence of the entire population. To resolve this, the mean position of the current population, denoted as K , is initialized. Individuals can shift toward the direction lying between the best individual, $V_{best}^{\tau }$ , and the mean position, K . Furthermore, even if the position of the current best individual, $V_{best}^{\tau }$ , remains unchanged for several generations, the mean position K evolves over generations, offering potential benefits in discovering improved solutions. Therefore, including the mean position K in the designed local exploitation strategy can improve the probability of partially avoiding local optima.
- The generalized standard variance, denoted as ${∁}_{j}$ , serves the purpose of augmenting the local search capability of the GNDO approach. Furthermore, based on Equations (12) and (13), generalized standard variances ${∁}_{j}$ can be considered as a random sequence utilized for performing local searches around the generalized mean position $\partial_{j}$ . Additionally, as per Equation (13), when the distance between the position of the jth individual, $V_{j}^{\tau }$ , and both the mean position, K , and the position of the best individual, $V_{best}^{\tau }$ , is greater, the variation in the generated random sequence becomes more pronounced. In other words, when the fitness value of an individual $V_{j}^{\tau }$ is very poor, there is a low likelihood of it discovering a better solution nearby. Consequently, a random sequence with pronounced fluctuation can assist such individuals in searching for improved solutions. Conversely, when an individual, $V_{j}^{\tau \;}$ possesses a good fitness value, there’s a higher probability of finding a better solution nearby. Therefore, a random sequence with less fluctuation can aid these individuals in achieving better solutions.
- The penalty factor, denoted as ∈ , plays a role in the GNDO algorithm by increasing the level of randomness in the generated generalized standard variance. Most penalty factors typically fall within the range of −1 to 1. It’s important to note that the generated generalized standard variances are always positive. Consequently, the penalty factor can expand the search directions of GNDO, thereby augmenting its search capability.

*Global exploration*. Global exploration involves systematically searching a solution space in order to identify promising regions that may contain valuable solutions. In GNDO, the global exploration is executed by utilizing a trio of randomly chosen individuals, a concept that can be formulated as follows:


(16) $$Z_{j}^{\tau }=V_{j}^{\tau }+\underset{Local\;Information\;Sharing}{\underset{︸}{\rho \;\times \left(\left|\gamma_{3}\right|\times Z_{1}\right)}}+\underset{Global\;Information\;Sharing}{\underset{︸}{\left(1-\rho \right)\times \left(\left|\gamma_{4}\right|\times Z_{2}\right)}}$$

Here, $\gamma_{3}$ and $\gamma_{4}$ are two random numbers following a standard normal distribution, and ρ, referred to as the adjustment parameter, is a random number within the range of 0–1. Additionally, there are two trail vectors denoted as $Z_{1}$ and $Z_{2}$ . Furthermore, the computation of $Z_{1}$ and $Z_{2}\;$ can be described as follows:


(17) $$Z_{1}=\{\begin{array}{c} V_{j}^{\tau }-V_{l1}^{\tau },\;if\;f\left(V_{j}^{\tau }\right)<f\left(V_{l1}^{\tau }\right)\\ V_{l1}^{\tau }-V_{j}^{\tau },\;\;\;\;\;\;\;Otherwise \end{array}$$

(18) $$Z_{2}=\{\begin{array}{c} V_{l2}^{\tau }-V_{l3}^{\tau },\;if\;f\left(V_{l2}^{\tau }\right)<f\left(V_{l3}^{\tau }\right)\\ V_{l3}^{\tau }-V_{l2}^{\tau },\;\;\;\;\;\;\;Otherwise \end{array}$$

where l1, l2, and l3  represent three randomly selected integers ranging from 1 to *n*, with the condition that l1≠l2≠l3≠j . Referring to Equations (7) and (8), the second term on the right-hand side of Equation (6) can be termed as the “local learning term,” signifying that solution p1 shares information with solution j . Similarly, the third term on the right-hand side of Equation (16) can be expressed as “global information sharing,” describing that individual j receives information from individuals l2 and l3 . The adjustment parameter, ρ , serves the purpose of balancing the two information-sharing strategies. Additionally, $\;\gamma_{3}$ and $\gamma_{4}$ are random numbers following a standard normal distribution, which extends the search space of GNDO during the global search process. The use of the absolute symbol in Equation (16) is maintained for consistency with the screening mechanism described in Equations (17) and (18).

*Novelty-proposed quantum mechanism.* Various techniques have been employed in selecting features. The metaheuristics have various shortcomings, including the unbalanced exploration and exploitation that impacts the algorithms’ ability to converge. Since optimization algorithms are typically used for feature selection, pattern recognition applications are expected to have excellent accuracy. Applying the quantum theory significantly enhances the typical GNDO optimization algorithm’s performance and accuracy. The initial population is generated with size N as:


(19) $$Z_{j}^{k}\left(k+1\right)=Z_{min}+r\times \left(Z_{max}-Z_{min}\right)\;$$

where $Z_{i}$ denotes the value of ith solution, r∈[0,1] denotes a random number, $Z_{max}$ and $Z_{min}$ represent maximum and minimum search space limits, and k is a current iteration of a feature space. The fitness is computed in the next step and determine the best solution based on the minimization function. To update the solution of the original GNDO algorithm, the following formulations have been employed.


(20) $${\text{Φ}}_{pd}=Z_{i,j}\left(k+1\right)$$

(21) $$=\{\begin{array}{c} {\text{Ω}}_{i}-\beta \times \left(G_{best}-Z_{i,j}\left(k\right)\right)\times ln\left(\frac{1}{u}\right),\;\;if\;t>0.5\\ {\text{Ω}}_{i}+\beta \times \left(G_{best}-Z_{i,j}\left(k\right)\right)\times ln\left(\frac{1}{u}\right),\;\;if\;t\leq 0.5 \end{array}$$

(22) $${\text{Ω}}_{i}=\theta +P_{best}+\left(1-\theta \right)*g_{best\left(i\right)}$$

(23) $$G_{best}=\frac{1}{N}\sum_{i=1}^{N}p\;Best_{i}$$

(24) $$\text{pd}\left(Z_{i,k+1}^{j}\right)=\frac{1}{L_{i,k}^{j}}\exp\left(-\frac{2\left|z_{i,k+1}^{j}-{ℵ}_{i,k}^{j}\right|}{L_{i,k}^{j}}\right)$$

The symbol ${\text{Ω}}_{i}$ denotes the local attractor, $P_{best}$ is best position of ith feature space, and ${ɡ}_{best\left(i\right)}$ is the best feature among the entire feature space. Updating the population is continued until the stop condition is not met. In the end, neural network classifiers are utilized to classify the chosen characteristics such as narrow neural networks and tri-layered neural networks. A feature vector of N×1,572 has been attained for this work.

A fitness function specified in Equation (25a) processes this procedure.


(28) $$F_{fc}=\left|\frac{f\left(\emptyset_{best}\right)}{f\left(\emptyset_{i}^{k}\right)}\right|$$

Every iteration, we take into account the average value of the chosen features, and the cost function is described as:


(26a) $$\emptyset_{cost}=\gamma_{\alpha }\times \delta_{error}+\gamma_{\beta }\times \left(\frac{num\_feature}{\text{max}\_feature}\right)$$

(27) $$\delta_{error}=1-\omega_{accuracy}$$

In the aforementioned equation, the coefficients are denoted by $\gamma_{\alpha }$ and $\gamma_{\beta }$ , with values of 0.99 and 0.01 for $\;\omega_{\alpha }\;$ and $\omega_{\beta }$ , respectively. The cost function is represented by $\emptyset_{cost}$ , and the accuracy derived from the fitness function is represented by $\omega_{accuracy}.$ Neural network classifiers are ultimately utilized to classify the final set of optimized features.

## 4 Experimental results and analysis

*Experimental setup.* Experiments were conducted using the INbreast dataset to classify breast cancer. The dataset was divided into a 50:50 split, with 50% of images from each class allocated for training and the remaining 50% for testing. A strong technique called cross-validation was employed to stop overfitting. A 10-fold cross-validation approach was employed for the testing results. Several hyperparameters have been selected in the training process, such as a momentum value of 0.776, epochs of 50, mini-batch size of 64, and stochastic gradient descent as an optimizer and learning rate value of 0.000241. All experiments were carried out on a computer with a Core i7 processor, 128GB of RAM, and a 12GB graphics card of RTX 3060 utilizing MATLAB2023a.

*Classifiers and performance measures.* For the classification task, multiple neural network classifiers were employed, including wide neural network (Wi-NN), medium neural network (Me-NN), narrow neural network (Na-NN), bi-layered neural network (Bi-NN), and tri-layered neural network (Ti-NN). The following metrics are used to calculate each classifier’s performance: accuracy (Acc), F1 score, precision rate, sensitivity rate, FNR, G-Measure, Kappa, and AUC. Every classifier’s time is likewise recorded in the test results.

The best performing classifier was identified by computing performance indicators across a variety of neural networks. There are 10 hidden layers and one fully connected layer in an Na-NN, 25 layers and one fully connected layer in an Me-NN, 100 hidden layers and one fully connected layer in a Wi-NN, 10 layers and two fully connected layers in a Tri-layered NN, and 10 layers and three fully connected layers in a Bi-NN.

*Experiments of the proposed framework.* The evaluation of the proposed framework has been performed based on the following experiments:

▪ Experiment 1—classification using a proposed four-block bottleneck network deep features.
▪ Experiment 2—classification using a proposed three-block bottleneck network deep features.
▪ Experiment 3—classification using a proposed features fusion approach.
▪ Experiment 4—classification utilizing the proposed quantum GNDO best feature selection technique.
▪ Comparison of the proposed framework accuracy and time with several neural nets such as Alexnet, VGG19, Resnet50, and a few more.
▪ Comparison among proposed quantum GNDO and few other optimization algorithms in terms of accuracy and time.

## 4.1 Proposed numerical results

**Figure 7:**
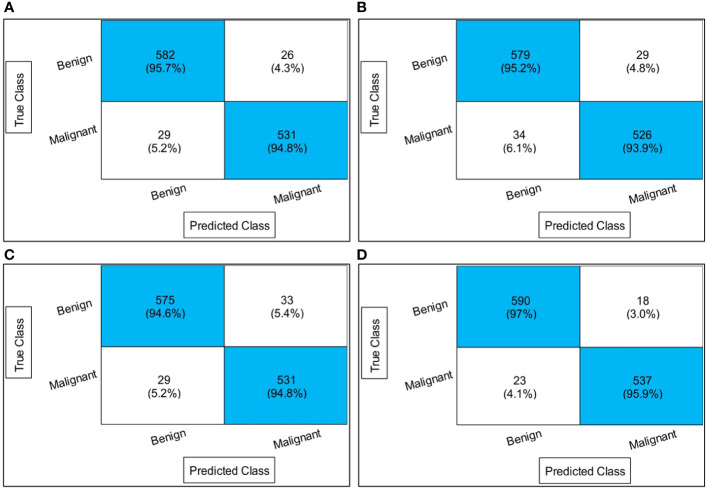
Confusion matrices of all experiments: **(A)** confusion matrix of experiment 1, **(B)** confusion matrix of experiment 2, **(C)** confusion matrix of experiment 3, and **(D)** confusion matrix of experiment 4.

**Table 1** presents the classification results using a proposed four-block bottleneck network deep features on the INbreast dataset. The top-performing classifier is the Wi-NN, achieving an impressive accuracy of 95.3%. This classifier also exhibits a sensitivity rate of 95.25%, precision rate of 95.3%, MCC of 90.57%, Kappa value of 90.56%, and F1 score of 95.49%, with a corresponding false negative rate (FNR) of 4.75%. The Tri-NN classifier scored the second-highest accuracy, which is 94.9%. Each classifier’s computational time is also recorded, and it is found that the medium NN classifier is computationally faster than the other classifiers on the list. The noted time of MNN classifier is 102.92 s, whereas the highest time of this experiment is 343.18 s. **Figure 7A** shows a confusion matrix of this experiment. In this figure, the correct prediction rate of a malignant class is 94.8%, whereas the FNR value was 5.2%.

**Table 1:** Classification results using a proposed four-block bottleneck network deep features on INbreast dataset.

Classifiers	Acc (%)	Sensitivity Rate (%)	Precision Rate %	F1 Score (%)	FNR(%)	MCC(%)	Kappa(%)	AUC	Time (s)
Na-NN	94.3	94.25	94.25	94.48	5.75	88.51	88.51	0.96	122.47
Me-NN	94.3	94.3	94.35	94.58	5.7	88.68	88.68	0.96	**102.92**
**Wi-NN**	**95.3**	**95.25**	**95.3**	**95.49**	**4.75**	**90.57**	**90.56**	**0.97**	343.18
Bi-NN	94.6	94.6	94.6	94.83	5.4	89.19	89.19	0.97	123.39
Ti-NN	94.9	94.85	94.85	95.07	5.15	89.71	89.71	0.97	176.51

Bold values denotes the best results.

**Table 2** presents the classification results using a proposed three-block bottleneck network deep features on INbreast dataset. The medium neural network (MNN) classifier is a top-performing classifier with an impressive accuracy of 94.6%. This classifier also shows a sensitivity rate of 94.55%, precision rate of 94.65%, MCC of 89.19%, Kappa value of 89.19%, and F1 score of 94.84%, with a corresponding FNR value of 5.45%. The WNN classifier achieved the second-highest accuracy of 94.1%. **Figure 7B** shows a confusion matrix of the MNN classifier that can be utilized to confirm the performance of MNN classifier. This figure shows that the correct prediction rate of a malignant class has been 93.9%. For every classifier, the computing time is also mentioned, and the minimum reported time of a bi-layered neural network is 43.705 s. In contrast, the wide-NN classifier has the highest consumed time of 181.96 s. Compared to experiment 1, the computational time of this experiment is slighter; however, the precision rate of the first experiment is better.

**Table 2:** Classification results using a proposed three-block bottleneck network deep features on INbreast dataset.

Classifiers	Acc (%)	Sensitivity Rate (%)	Precision Rate %	F1 Score (%)	FNR(%)	MCC(%)	Kappa(%)	AUC	Time (s)
Na-NN	93.3	93.35	93.3	93.60	6.65	86.62	86.62	0.95	61.901
**Me-NN**	**94.6**	**94.55**	**94.65**	**94.84**	**5.45**	**89.19**	**89.19**	**0.98**	87.218
Wi-NN	94.1	94.15	94.05	94.29	5.85	88.18	88.17	0.98	181.96
Bi-NN	92.3	92.3	92.3	92.60	7.7	84.56	84.56	0.96	**43.705**
Ti-NN	93.4	93.4	93.45	93.68	6.6	86.79	86.79	0.96	59.943

Bold values denotes the best results.

**Table 3** describes the classification results of the proposed feature fusion. In this experiment, the features of the proposed three- and four-block bottleneck have been fused and the numerical results computed. The top-performing classifier of this experiment is wide neural network, which achieved an impressive accuracy of 94.7%, which a confusion matrix can confirm, as shown in **Figure 7C**. This classifier also shows a sensitivity rate of 94.7%, precision rate of 94.65%, MCC of 89.37%, Kappa of 89.37%, and F1 score of 94.88%, with a corresponding FNR of 5.3%. Compared with **Table 2**, the performance of this experiment has been improved; however, the performance of **Table 1** (experiment 1) has been better. Moreover, the computational time of the fusion process is better than that of experiments 1 and 2. The fusion process’ minimum and highest noted computational time is 31.923 (s) and 41.374 (s), respectively.

**Table 3:** Classification results using the proposed fusion of three-block bottleneck network and four-block bottleneck deep features.

Classifiers	Acc (%)	Sensitivity Rate (%)	Precision Rate %	F1 Score (%)	FNR(%)	MCC(%)	Kappa(%)	AUC	Time (s)
Na-NN	94.3	94.25	94.3	94.52	5.75	88.51	88.50	0.96	34.417
Me-NN	94.3	94.25	94.25	94.49	5.75	88.51	88.51	0.98	**31.923**
**Wi-NN**	**94.7**	**94.7**	**94.65**	**94.88**	**5.3**	**89.37**	**89.37**	**0.98**	41.374
Bi-NN	93.7	93.65	93.65	93.88	6.35	87.32	87.31	0.95	33.409
Ti-NN	93.1	93.05	93.05	93.32	6.95	86.11	86.11	0.95	40.857

Bold values denotes the best results.

Finally, the proposed quantum GNDO-based feature selection technique was applied to the fused feature vector, and the best features were selected. Neural network classifiers are provided with best-selected information in order to improve classification accuracy. **Table 4** shows the classification results of the proposed quantum GNDO feature selection technique, which obtained the best accuracy of 96.5% using a bi-layered neural network classifier. This classifier’s other listed performance measures are sensitivity rate of 96.45%, precision rate of 96.5%, MCC of 92.97%, Kappa of 92.97%, and F1 score of 96.64%. The Me-NN classifier got the second best accuracy of 96.2%. The confusion matrix of the Wi-NN classifier is shown in **Figure 7D**, which can be utilized to confirm the overall computed performance measures. This figure shows that the malignant class correct prediction rate is 95.9%, better than the previous three experiments. Every classifier’s computation time has been recorded, and the Bi-NN classifier demonstrates a relatively shorter execution time (5.9234 s) compared to the other classifiers. Compared to the performance of the recent three experiments, the feature selection technique improved the computational time. It increased the overall performance of the proposed framework (i.e., accuracy, precision, Kappa, G measure).

**Table 4:** The final classification results utilizing the proposed quantum GNDO based best feature selection technique.

Classifiers	Acc (%)	Sensitivity Rate (%)	Precision Rate %	F1 Score (%)	FNR(%)	MCC(%)	Kappa(%)	AUC	Time (s)
Na-NN	96.1	96.05	96.1	96.24	3.95	92.11	92.11	0.97	6.4629
Me-NN	96.2	96.2	96.25	96.39	3.8	92.45	92.45	0.97	6.4024
Wi-NN	95.8	95.75	95.85	96.01	4.25	91.61	91.59	0.98	10.089
**Bi-NN**	**96.5**	**96.45**	**96.5**	**96.64**	**3.55**	**92.97**	**92.97**	**0.98**	**5.9234**
Ti-NN	96.1	96.05	96.2	96.34	3.95	92.30	92.27	0.97	6.1177

Bold values denotes the best results.

### 4.2 Discussion

**Figure 8:**
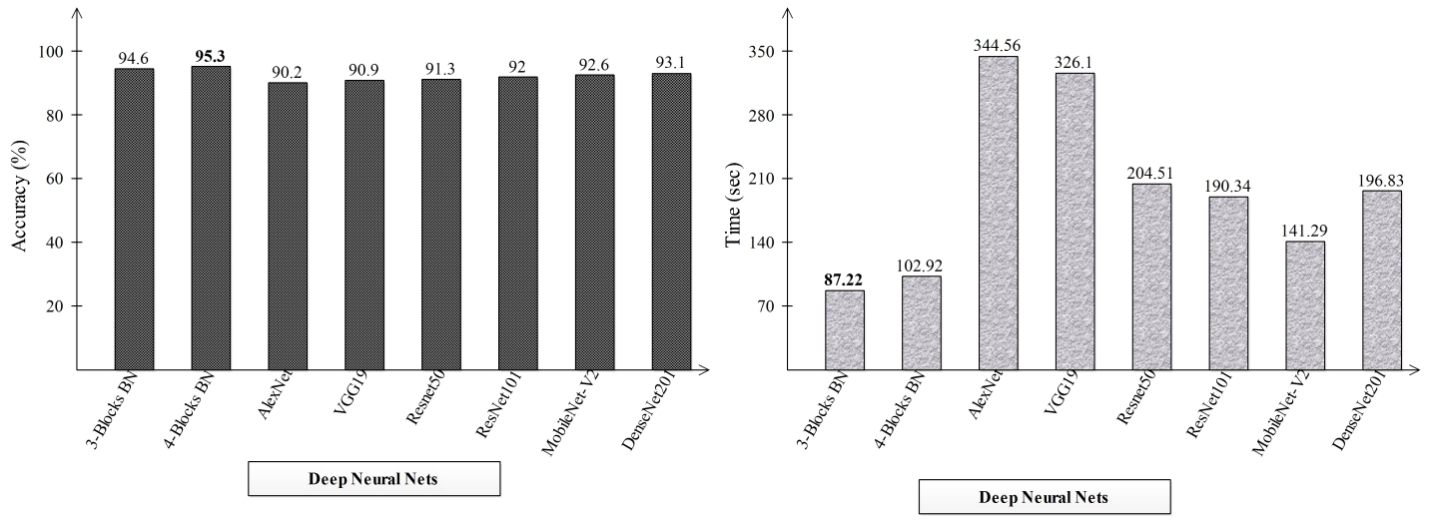
Comparison of proposed deep neural nets with pre-trained deep neural networks in terms of accuracy (%) and time (s).

2This section explains the suggested framework along with a thorough discussion of it. In the first stage, the accuracy and computation time of the suggested framework’s performance were compared to those of many neural nets. Alexnet, VGG19, Resnet50, ResNet101, MobileNet-V2, and Densenet201 are the neural nets that were chosen. The accuracy and time-based comparison are displayed in **Figure 8**. The accuracy-based comparison of several deep neural networks, including pre-trained and proposed three- and four-block BN networks, is displayed on the left side of this image. The proposed four-block bottleneck reached the highest accuracy of 95.3% bottleneck CNN design (four-block BN), while 94.6% was obtained by the second proposed architecture, three-block bottleneck (three-block BN). The accuracy of the remaining pre-trained models was 90.2, 90.9, 91.3, 92, 92.6, and 93.1%, in that order. A time-based comparison is also made, and it is found that because of their bigger parameters, the AlexNet and VGG19 models require the longest runs, 344.56 (s) and 326.1 (s), respectively. The suggested three-block BN architecture completed its execution in 87.22 s at the very least, and the four-block BN architecture took 102.92 s.

**Figure 9:**
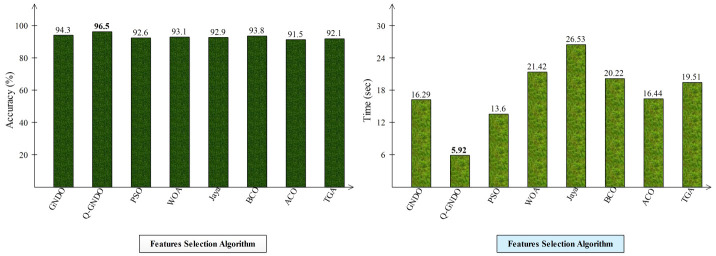
Comparison of proposed quantum GNDO features selection technique with several selection algorithms in terms of accuracy (%) and time (s).

Different optimization algorithms, including the original GNDO, the proposed quantum GNDO, PSO, Whale Optimization, Jaya Optimization, BCO, Ant Lion Optimization, and Tree Growth Optimization, are compared in **Figure 9**. Each technique is substituted for the suggested quantum GNDO algorithm in **Figure 1**, and the outcomes (accuracy and time) are computed. This graphic displays the accuracy plot on the left and the time plot on the right. By analyzing this left-side plot, the Q-GNDO algorithm’s accuracy is higher than that of all the other specified methods. Furthermore, the graphic on the right side demonstrates that the computing time of the suggested GNDO algorithm is lower than that of the other feature selection methods.

*Student’s t-test analysis.* A statistical test called the Student’s t-test is carried out to contrast the means of two independent groups and analyze if there is a difference between them that is statistically significant. We initially selected two classifiers, Me-NN and Ti-NN, based on the highest and lowest accuracy. Then, we define a hypothesis that $h_{o}$ = there is no significant difference in the accuracy of the selected classifiers. **Table 5** describes the accuracies of selected classifiers. Initially, the difference is computed, as shown in this table, and the mean value is 0.775. After that, a standard deviation is computed, and a value of 0.531 is obtained by employing Equation 28.

**Table 5:** Selected classifiers for the evaluation of Student’s t-test.

Classifiers	Four-Bottleneck Block Model	Three-Bottleneck Blocks Model	Fusion	Generalized Normal Distribution Optimization
Medium NN	94.3	94.6	94.3	96.2
Tri-layered NN	94.9	93.4	93.1	96.1
**Difference**	**0.6**	**1.2**	**1.2**	**0.1**


(28) $$Standard\;Deviation=\sigma =\sqrt{\frac{\sum_{i=1}^{N}{\left(Diff_{i}-\mu \right)}^{2}}{N-1}}$$

By employing the value of mean and standard deviation, the final t value is computed by the following equation by **Equation 29**:


(29) $$t-selection=t=\frac{\sqrt{N}\times \mu }{\sigma }$$

By this equation, the obtained value of t is 2.919, which is finally employed for the confidence interval analysis. We checked the value of t at p=0.05, and the returned value from the t-table is (−3.182, +3.182), which shows that the value of t falls under this interval. Hence, our hypothesis has been accepted.

**Figure 10:**
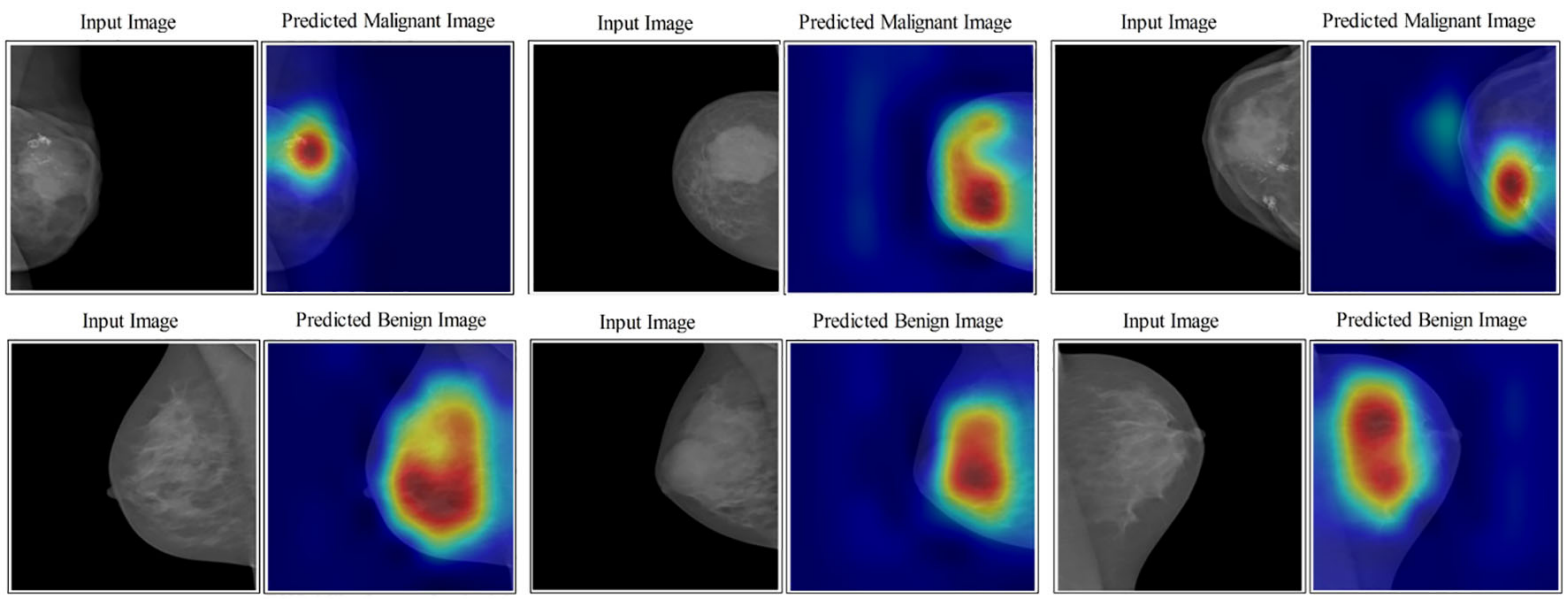
Lesion localization results based on proposed four- and three-block bottleneck architectures using GradCAM.

In the last step, an indirect comparison of the proposed framework’s accuracy with the recently published methods has been conducted, as shown in **Table 6**. In this table, the authors in (30) obtained an accuracy of 83.19%, later improved by authors in (42) at 95.6%. The other listed methods in this table, such as (43–45), obtained accuracies of 95.1%, 93.0%, and 96.0%, respectively. The proposed method obtained an accuracy of 96.5%, which is better than the recently presented methods. In addition, for the proposed method, the sensitivity rate is 96.45, the precision rate is 96.5, the F1 score value is 96.64, the MCC value is 92.97%, and the Kappa value is 92.97%, respectively. **Figure 10** illustrates the visual results of the proposed architecture using the GradCAM technique. The GradCAM is utilized for the localization of cancer regions (malignant) after employing the deeper information of the last convolutional layer. A red-to-blue scale is typically used in Grad-CAM heatmaps, with red denoting the most relevant and blue the least important. The color’s intensity indicates the level of significance. Moreover, benign label images are also shown in this image for visualization.

**Table 6:** Comparison of the proposed architecture accuracy with recent techniques.

References	Year	Dataset	Method	Accuracy (%)
(30)	2023	INbreast	CAD methodology	83.19
(42)	2023	INbreast	Feature Selection and Enhancement network (FSE-Net)	95.6
(43)	2023	INbreast	computer Aided Diagnosis	95.1
(44)	2023	INbreast	ResNet-50 convolutional neural network	93.0
(45)	2022	INbreast	TwoViewDensityNet, an end-to-end deep learning-based method	96.0
**Proposed**		INbreast		Accuracy=96.5%, Sensitivity Rate= 96.45, Precision rate=96.5, F1 Score=96.64

## 5 Conclusion

In this proposed work, two novel deep learning architectures (four- and three-block bottleneck) have been proposed along with a novel Kernel CCA fusion and quantum GNDO optimization for the improved classification accuracy of malignant and benign breast cancer. The proposed architectures consist of a few parallel blocks and a single bypass layer that improved the learning of a model on the selected mammogram images. Bayesian Optimization is employed to initialize hyperparameters of both architectures and train up to 100 epochs. Features are extracted from the deeper layers and fused using a novel Kernel CCA approach. Only important features that improved the accuracy and precision rate of the proposed framework compared to the individual proposed architectures are fused in this step. In addition, to make the proposed framework more efficient, we proposed a novel quantum GNDO optimization algorithm that selects the best features. The selection process improved the accuracy to 96.5% and considerably reduced the computational time. The proposed framework is compared with state-of-the-art (SOTA) techniques and achieves an enhanced accuracy.

*Clinical challenge and future directions*. In clinical practice, this proposed architecture can face the following challenges: i) a higher amount of training data is required; ii) high-computational computers are required; and iii) an AI expert is required in the clinic to evaluate the output. There are a few limitations of this work that can be considered as an improvement in the future. The one major limitation of this work is deeper layer feature extraction and fusion of these features instead of fusion within the network. The out-of-the-network fusion process consumed extra time. A self-attention and vision transformer network will be designed to fuse the information for improved accuracy and less computational time.

## Data availability statement

The original contributions presented in the study are included in the article/supplementary material, further inquiries can be directed to the corresponding authors.

## Author contributions

KJ: Conceptualization, Data curation, Methodology, Software, Writing – original draft. MK: Conceptualization, Methodology, Software, Supervision, Writing – original draft. MH: Conceptualization, Investigation, Methodology, Software, Writing – original draft. OA: Conceptualization, Formal Analysis, Methodology, Project administration, Resources, Software, Writing – review & editing. MT-H: Formal Analysis, Funding acquisition, Investigation, Methodology, Resources, Validation, Visualization, Writing – review & editing. AM: Funding acquisition, Methodology, Project administration, Supervision, Visualization, Writing – original draft.
